# Comprehensive genetic diversity and genome-wide association studies revealed the genetic basis of avocado fruit quality traits

**DOI:** 10.3389/fpls.2024.1433436

**Published:** 2024-08-13

**Authors:** Jin Li, Shamseldeen Eltaher, Barbie Freeman, Sukhwinder Singh, Gul Shad Ali

**Affiliations:** Subtropical Horticulture Research Station, United States Department of Agriculture, Agriculture Research Service, Miami, FL, United States

**Keywords:** avocado germplasm, genomic diversity, population structure, SNP markers, genome-wide association study (GWAS), fruit quality traits

## Abstract

**Introduction:**

Avocado (*Persea americana*) is a highly nutritious fruit gaining worldwide popularity. However, its cultivation is currently reliant on a limited number of cultivars with restricted genetic diversity. This study aims to investigate the genetic diversity and population structure of avocado germplasm and identify genetic loci associated with key fruit quality traits that influence customer preference.

**Methods:**

A diversity panel of 110 avocado accessions was analyzed using 4,706 high-quality single nucleotide polymorphisms (SNPs). Genetic diversity and population structure were analyzed using pairwise FST, AMOVA, admixture analysis, and phylogenetic analysis. Genome-wide association studies (GWAS) were conducted targeting nine fruit quality traits using two models: General Linear Model (GLM) with Principal Component Analysis (PCA) and Mixed Linear Model (MLM) with PCA and kinship (PCA + K).

**Results:**

The analysis revealed three distinct populations corresponding to the three avocado ecotypes: Guatemalan, West Indian, and Mexican. Phylogenetic analysis indicated a closer relationship between the Guatemalan and West Indian races compared to the Mexican race in our Florida germplasm collection. GWAS led to identification of 12 markers within 11 genomic regions significantly associated with fruit quality traits such as fruit color, shape, taste, and skin texture. These markers explained between 14.84% to 43.96% of the phenotypic variance, with an average of 24.63%. Annotation of these genomic regions unveiled candidate genes potentially responsible for controlling these traits.

**Discussion:**

The findings enhance our understanding of genetic diversity and population structure in avocado germplasm. The identified genetic loci provide valuable insights into the genetic basis of fruit quality traits, aiding breeding programs in developing improved avocado cultivars. Marker-assisted selection can accelerate the development of new varieties, promoting a more diverse and resilient avocado market.

## Introduction

Avocado, scientifically known as *Persea americana* Mill., is an evergreen tree belonging to the Lauraceae family. It is indigenous to Mexico, Central America, and South America (Silva and Ledesma, 2014). The fruit of the avocado tree is highly nutritious due to its rich content of monounsaturated fats and various vitamins (Alvizouri-Muñoz et al., 1992). Research has shown that consuming avocados as a substitute for unhealthy fats over a long period can significantly reduce the risk of cardiovascular diseases (Pacheco et al., 2022).

Avocado cultivation has grown globally, with significant production in Mexico, Columbia, and Peru (Denvir et al., 2021). In 2022, American avocado consumption exceeded 2.7 billion pounds, indicating that avocados are in high demand (Shahbandeh, 2023). Avocado is an economically significant crop in many countries and provides employment opportunities throughout the supply chain (Williams et al., 2017). Its versatility and nutritional value have made avocados a popular ingredient in various cuisines worldwide, further cementing their significance in global food culture.

Avocado is classified into three ecotypes: Guatemalan (*P. americana* var. *guatemalensis* L. Wms.), Mexican (*P. americana* var. *drymifolia* Schlecht. et Cham. Blake), and West Indian (*Persea americana* var. *americana*) (Bergh and Ellstrand, 1986; Chen et al., 2008). These races originated from distinct geographical regions: West Indian from Mesoamerican coastal lowlands, Guatemalan from Guatemalan highlands, and Mexican from Mexican highlands (Gazit and Degani, 2002). Morphologically, Mexican avocados tend to be smaller in size with larger seeds, thin skins, and a waxy bloom texture. In contrast, Guatemalan and West Indian avocados exhibit variable fruit sizes, with Guatemalans having thick and rough skins and smaller seeds. Additionally, Mexican avocados are notably rich in oil content, whereas West Indian fruits generally have lower oil levels (Bergh and Ellstrand, 1986; Scora et al., 2002; Ayala-Silva et al., 2019). These ecotypes were introduced to the United States in the mid-to-late 19th century. They underwent extensive hybridization, developing interracial hybrids with promising characteristics, which are widely used in commercial avocado cultivation today (Schaffer et al., 2013).Over the years, horticultural characteristics of avocados have exhibited extensive variation, primarily due to cross-pollination and sexual propagation. Understanding genetic diversity is crucial for collecting, preserving, and utilizing avocado germplasm in crop development. Molecular markers, known for their high polymorphism and heritability, play a pivotal role in characterizing the genetic makeup of germplasm (Barcaccia, 2009). These markers are particularly advantageous for early identification of accessions and varieties with desirable traits (Talavera et al., 2019). This accelerates the breeding process for fruit trees such as avocados significantly, which have a lengthy juvenile period of up to 15 years before initial blooming and fruit production (Gazit and Degani, 2002). Various molecular markers, including random amplified polymorphic DNA (RAPD) (Chang et al., 2003; Jc et al., 2016), restriction fragment length polymorphism (RFLP) (Davis et al., 1998), and Simple Sequence Repeats (SSRs) (Mhameed et al., 1997; Ashworth and Clegg, 2003; Schnell et al., 2003; Gross-German and Viruel, 2013; Álvarez et al., 2020; Sanchez-Gonzalez et al., 2020), have been widely employed to analyze the genetic diversity of avocado accessions worldwide. However, earlier research on the three ecological races - West Indian, Mexican, and Guatemalan - yielded inconsistent results. While some studies grouped Guatemalan and West Indian races based on morphological traits and early DNA markers like RAPD and RFLP (Kopp, 1966; Mhameed et al., 1997; Fiedler et al., 1998; Ashworth and Clegg, 2003), others suggested a closer relationship between Guatemalan and Mexican races (Gross-German and Viruel, 2013; Ge et al., 2019; Wienk et al., 2022; Berdugo-Cely et al., 2023).

Conventional molecular markers have encountered challenges in accurately distinguishing among avocado genomes, particularly those with subtle differences resulting from recent interracial hybridization (Ashworth and Clegg, 2003). However, with advancements in high-throughput technologies such as microarray, next-generation sequencing (NGS) and genotyping-by-sequencing (GBS), single-nucleotide polymorphisms (SNPs) have emerged as powerful genetic markers (Poland and Rife, 2012; He et al., 2014; Ophir et al., 2014; Sherman et al., 2015; Rubinstein et al., 2019). These common genetic variations enable comprehensive genome-wide variability estimation (Mammadov et al., 2012; Morgil et al., 2020). Meanwhile, molecular biology methodologies, including Quantitative Trait Locus (QTL) mapping and Genome-Wide Association Studies (GWAS), have demonstrated considerable potential in identifying markers and candidate genes associated with important traits such as yield, disease resistance, and stress tolerance (Brachi et al., 2011; Pliego-Alfaro et al., 2013; Alseekh et al., 2021; Tibbs-Cortes et al., 2021). Furthermore, a chromosome-scale genome assembly of the most popular avocado cultivar ‘Hass’ has been published and revealed a diploid genome with a chromosome number of 2*n* = 24 and an approximate genome size of 920 Mb (Nath et al., 2022). This genomic information serves as a valuable reference for identifying genetic foundations underlying desirable avocado traits.

Fruit traits, such as color and shape, exert significant influence on consumer preferences, market acceptance, and the overall commercial viability of agricultural produce. Moreover, characteristics such as fruit skin texture can profoundly affect post-harvest handling, storage, and transportation practices. GWAS has been widely employed to investigate fruit quality traits. For instance, a study involving 312 sand pear (*Pyrus pyrifolia*) accessions explored loci associated with 11 agronomic traits, including fruit skin color and days to fruit development (Zhang et al., 2021). The *PbrSTONE* gene was identified and functionally validated for its involvement in regulating stone cell formation (Zhang et al., 2021). Another study, focusing on 129 peach (*Prunus persica* L.) accessions, investigated SNPs associated with 10 qualitative traits, such as fruit shape, flesh texture, and flesh color (Cao et al., 2016). Similar GWAS and population structure studies have been reported in other tree crops, including apples (*Malus x domestica* Borkh) (Urrestarazu et al., 2017; McClure et al., 2019), grapes (*Vitis vinifera* L.) (Guo et al., 2019; Flutre et al., 2022), and Ginkgo (Hu et al., 2023). Based on these findings, breeders can make informed decisions when selecting parental lines and designing breeding strategies to enhance the efficiency and success of crop improvement programs (Varshney et al., 2021). Despite the considerable significance of fruit quality traits, limited attention has been devoted to improve these characteristics in avocados. There is an urgent need for increased efforts in this area.

In previous studies, a set of 5,050 high quality SNP markers was developed using transcriptome data obtained through Illumina sequencing technology (Kuhn et al., 2019a). We aligned these markers to the recently published ‘Hass’ reference genome (GCA_029852735.1) (Nath et al., 2022) resulting in 4,706 valid SNPs. These makers were used to assess the genetic diversity and population structure of 110 *Persea* accessions. Concurrently, correlations between these SNPs and nine fruit quality traits, collected from 2017 to 2022, including fruit skin color, fruit shape, fruit taste, and fruit skin texture, were examined. Significant associations between genomic regions and fruit quality traits were detected, leading to the identification of candidate genes through functional inference. These findings hold promise for marker-assisted selection in avocado breeding, potentially reducing the time and financial resources required for the development and release of new avocado varieties. Furthermore, these findings represent an invaluable resource for elucidating gene functions and the intricate molecular mechanisms underlying fruit quality traits. By leveraging these markers, researchers in the future can unravel genetic pathways governing fruit development and quality, leading to insights applicable to a wide range of crops and advancing crop genetics and improvement efforts on a global scale.

## Materials and methods

### Germplasm collection and growth conditions

This study was conducted at the Subtropical Horticulture Research Station in Miami, FL, part of the United States Department of Agriculture’s Agriculture Research Service. The location contains 167 unique *Persea* sp. accessions, and germplasm information is available on the GRIN-Global website (www.ars-GRIN.gov). The average temperature was 75.60°F, ranging from 62°F to 90°F, with an average annual precipitation of 60.51 inches (USA.com). A subset of 110 accessions was used for fruit quality evaluation, as detailed in **Supplementary Table 1.**

### Phenotypic evaluation

Power analysis determined that a sample size of 12 was necessary for each accession (Ryan, 2013). During six years, from 2017 to 2022, around 12 to 36 fruits per accession were collected and evaluated for several characteristics during the ripening process, including skin color, skin texture, fruit shape, and fruit taste (**Supplementary Table S2**). The standards for ripening varied among the different varieties, which included the darkening of the peel color, softening of the fruit, and formation of craters at the connection between the stem and the fruit.

The classification of fruit traits was as follows: Skin color - green, green-brown, green-black, red-brown, purple, black, and speckled; Fruit shape - oblate, spheroid, high spheroid, ellipsoid, narrowly obovate, obovate, pyriform, clavate, and rhomboidal; Fruit taste - bitter, nutty, bland, watery, creamy, and buttery; Skin texture - smooth, rough, and very rough. The phenotypic evaluation criteria were referenced from the information provided by the University of California, Riverside, Avocado Variety Collection website (https://avocado.ucr.edu/fruit-colors-and-shapes).

The phenotypic data were converted into scales for the GWAS analyses. The color was evaluated based on two aspects: greenness and redness. For greenness, the scale ranged from light to dark with four categories: 1 for green, 2 for partially green, including green-brown, green-black, and speckled, 3 for red-brown and purple, and 4 for black. In assessing redness, the three categories were defined as follows: 1 for red-brown, 2 for red-related colors such as green-brown and purple, and 3 for all other red-unrelated colors. Skin texture was evaluated on a scale of smooth (1), rough (2), and extra rough (3). Taste was simplified into presence or absence for each taste category: 1 for present and 2 for absent. The Spearman’s rank correlation coefficients (*ρ*) between pairs of avocado fruit quality traits were calculated using the cor function in R v. 4.3.1 (R Core Team, 2021) and visualized by ggplot2 v. 3.4.4 (Wickham, 2016).

### DNA isolation, sequencing, and genotyping

DNA was isolated from the entire SHRS germplasm collection, including the selected set of 110 avocado accessions, using the FastPrep method (MPBio, Santa Ana, CA), and genotyped using the Illumina Infinium II 6000 SNP chip (Illumina, San Diego, CA, USA), essentially as described before (Kuhn et al., 2019b). Previously, 5,050 high-quality SNP markers were created using transcriptome data from different common varieties of avocado, namely ‘Hass,’ ‘Bacon,’ ‘Simmonds,’ and ‘Tonnage’ (Kuhn et al., 2019a). To update the marker coordinates, the 121-bp contigs centered on each SNP marker were mapped to the latest avocado ‘Hass’ reference genome (GCA_029852735.1) (Nath et al., 2022) using Burrow-Wheeler Aligner v. 0.7.17 (Li and Durbin, 2009) (**Supplementary Table S3**). To perform functional annotation on the coding sequence of the ‘Hass’ reference genome, eggNOG-mapper v.2.1.12 with the Pfam, GO, and KEGG databases (Ashburner et al., 2000; Mistry et al., 2020; Cantalapiedra et al., 2021; Kanehisa et al., 2022; Aleksander et al., 2023) was used. Furthermore, a custom Python code was used to annotate the SNP markers to include all the gene information within a 200 kb window centered on each SNP.

The SNPs were filtered further to eliminate loci with a minor allele frequency (MAF) less than 0.01 and a missing SNP genotype rate exceeding 20% using TASSEL 5.0 (Bradbury et al., 2007). This resulted in 4,706 SNPs, which were used for downstream analyses. VCFtools v. 0.1.16 (Danecek et al., 2011) was employed for statistical summarization of SNPs. Non-synonymous and synonymous SNPs were identified, and the non-synonymous/synonymous (ω =*Ka/Ks*) ratios were calculated, as previously described (Kuhn et al., 2019b).

### Phylogenetics, population structure analysis, and analysis of molecular variance

The alleles present at 4,706 loci were concatenated and aligned using MAFFT v. 7.505 (Katoh and Standley, 2013). Then, a maximum-likelihood (ML) phylogenetic tree was created using the GTRGAMMA model in RAxML v. 8.2.12 (Stamatakis, 2014) supported by 100 bootstrap replicates. The tree was visualized using FigTree v1.4.4 (http://tree.bio.ed.ac.uk/software/figtree/). The avocado race information was color-coded for better understanding.

Population structure analysis was conducted on 4,706 SNPs using ADMIXTURE v. 1.3.0 (Alexander et al., 2009), with genetic cluster numbers (*K*) ranging from 1 to 9. The optimal population numbers were determined through the cross-validation error rate calculation. The population structures at *K* = 3, 5, and 7 were visualized using the R package ggplot2 v.3.4.4. Genetic divergences between populations were estimated by calculating the pairwise fixation index (*F*_ST_) using VCFtools v. 0.1.16 (Danecek et al., 2011) with the method of Weir and Cockerham (1984) based on the 4,706 SNP markers in 10 kb windows. High *F*_ST_ values indicated a significant degree of genetic differentiation between populations. For the analysis of molecular variance (AMOVA), the number of subpopulations identified based on ADMIXTURE results was utilized. Genetic indices, including expected heterozygosity (*H*_E_) and Nei’s unbiased measures of genetic identity, were calculated using GeneAlEx v. 6.41 (Peakall and Smouse, 2006). The software was also used for AMOVA and genetic index estimates.

Principal Component Analysis (PCA) was conducted on the top 10 components using PLINK 2.0 (Chang et al., 2015) for 110 avocado accessions. The first two principal components were visualized using ggplot2 v. 3.4.4. To measure the degree of linkage disequilibrium (LD), the squared allele frequency (*r* ^2^) and the distance between the 4,706 SNP pairs were calculated using PopLDdecay v3.42 (Zhang et al., 2018). An LD decay plot was generated using the Plot_MutiPop.pl script with LD estimates plotted against physical distance (kb) in three avocado populations. The *r* ^2^ values were averaged in short-distance windows of 1000 bp and long-distance windows of 20,000 bp, with a breakpoint at 10,000 bp.

### Genome-wide association studies and pathway enrichment analyses

To address population stratification in the GWAS analyses, Centered Identical by State (IBS) kinship using a maximum of 6 alleles and PCA with the top 5 components were calculated. The association between 4,706 SNPs and fruit quality traits in 110 avocado accessions were assessed using two models: Generalized Linear Models (GLM) with PCA (subjected to 1000 permutations) and Mixed Linear Models (MLA) with PCA + Kinship in TASSEL version 5.0. A Bonferroni correction threshold of -log_10_(0.05/number of SNPs) set at 4.97 was used to identify significant SNPs associated with fruit quality traits. Additionally, *p*-values thresholds of 0.0001 and 0.0005 for slightly weaker associations were considered to prevent overlooking functional regions. The GWAS results including QQ-plots (quantile-quantile plots) to mitigate false positives by assessing the distribution of *p*-values were visualized by the CMplot package (Yin et al., 2021) in R v. 4.3.1.

The LD *r* ^2^ value of 0.2 was utilized as a moderate threshold for linkage with SNPs within 200 kb window commonly exhibiting stronger associations than the threshold. This method was also employed in Dujak et al. (2023) while investigating fruit size and shape traits in apple genotypes. All genes within 200 kb windows centered on markers associated with fruit quality traits were identified. Then, gene ontology (GO) and Kyoto Encyclopedia of Genes and Genomes (KEGG) pathway enrichment analyses were performed on these genes utilizing an annotated gene set from the ‘Hass’ genome as reference (Nath et al., 2022), and the results were compared with the plant database in TBtools 2 (Chen et al., 2023). The resulting data were filtered, and the top GO terms and KEGG pathways with high enrichment scores and significant *p*-value were plotted with custom R code with ggplot2.

## Results

### Phenotypic variation of fruit quality traits

**Figure 1:**
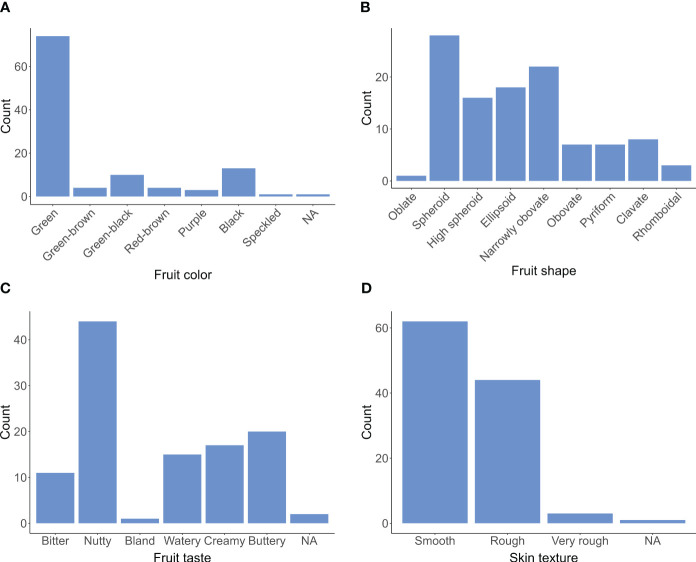
Histogram displaying the distribution of fruit quality traits, including color **(A)**, shape **(B)**, taste **(C)**, and skin texture **(D)**, from 110 avocados (*P. americana*) collections gathered between 2017 and 2022.

The avocado collection exhibits remarkable diversity, as evidenced by the significant variation observed in the fruit quality traits. The overall distribution of data is shown in **Figure 1**. These findings suggest that the phenotypic data are suitable for GWAS analysis, aiding in identifying specific genetic contributions to each trait and mitigating confounding factors.

Of all the avocado accessions, 67.3% had pure green fruits, 13.6% had green mixed with brown or black, and approximately 18.2% had non-green colors, including black, purple, and red-brown. Most fruit shapes were spheroid or obovate, but 16.4% had uncommon shapes like pear, clavate, or rhomboidal. Regarding taste, 40.0% of avocado accessions had a nutty flavor, 13.6% were watery, and 10.0% had a bitter taste. 56.4% had smooth skin for skin texture, while the rest had rough skin. We also observed an association between skin texture and ecotypes, revealing distinct patterns among avocado accessions. Specifically, Guatemalan accessions exhibited predominantly rough or very rough skin texture, with 18 out of 19 accessions falling into this category. In contrast, 12 out of 14 Mexican accessions displayed smooth skin. The West Indian accessions, however, demonstrated a diverse mixture of skin texture types. These findings align with previous studies (Ashworth and Clegg, 2003; Ashworth and Rolshausen, 2016).

**Figure 2:**
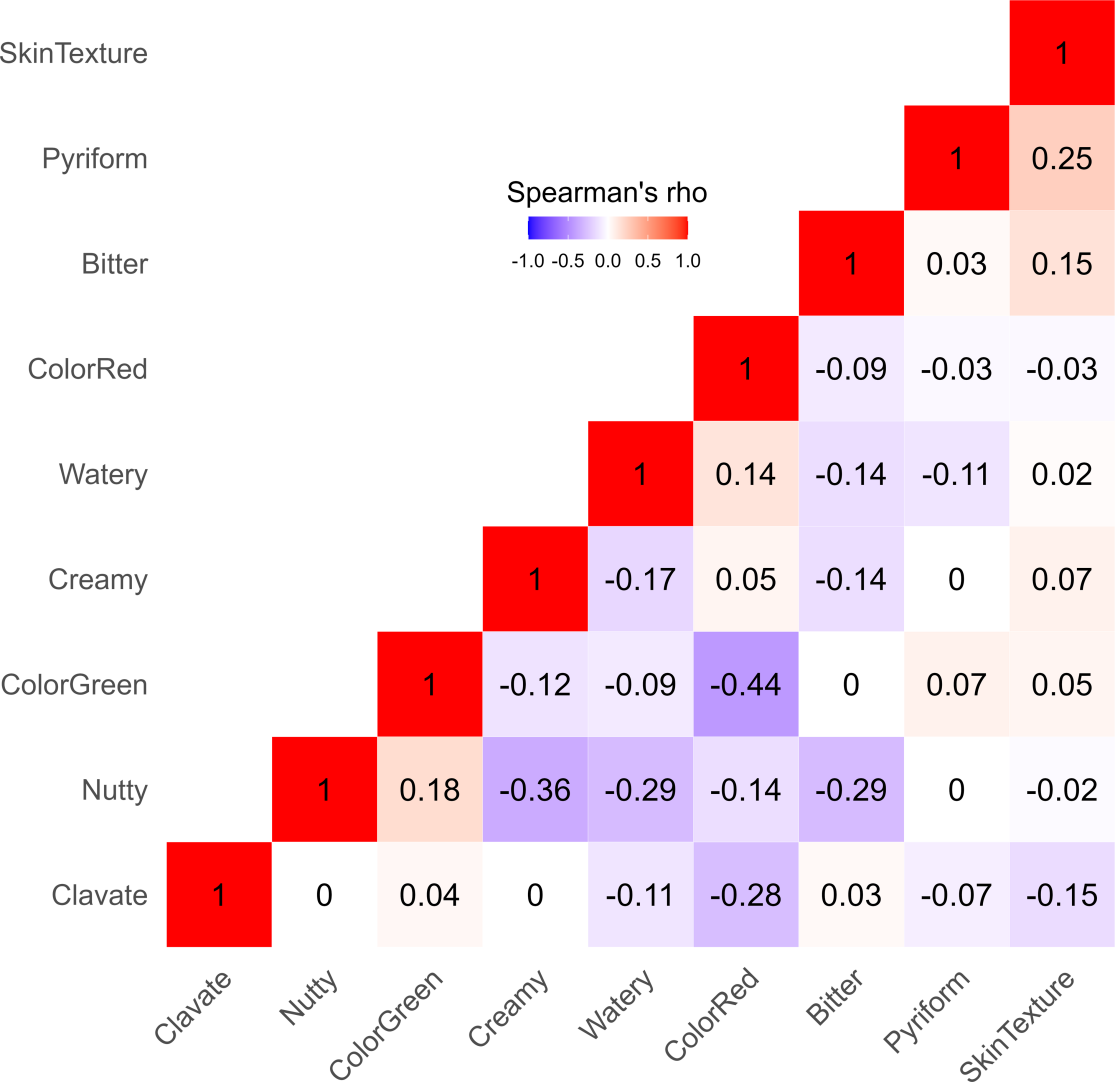
Heatmap displaying the Spearman’s correlation coefficients (*ρ*) between various fruit quality traits for 110 avocados (*P. americana*) accessions. Each square in the heatmap grid is colored to represent the strength of correlation between two traits - red for positive correlation, blue for negative correlation, and white for low correlation.

Most fruit quality traits had a weak or no correlation with each other, as shown by Spearman’s correlation coefficients (*ρ*) ranging between -0.44 and 0.25 (**Figure 2**). However, moderate negative correlations were found between nutty and creamy tastes *(ρ* = -0.36), nutty and watery tastes (*ρ* = -0.29), and nutty and bitter tastes (*ρ* = -0.29). As anticipated, skin color ratings that ranked fruit according to the degree of redness were negatively correlated with green skin color ratings (*ρ* = -0.44).

### Population structure and phylogenetic analyses reveal genetic relationships among avocado varieties

In this study, 5,050 SNP markers previously reported and designed based on the transcriptome data (Kuhn et al., 2019b) were utilized. We remapped these markers to the latest ‘Hass’ reference genome (GCA_029852735.1), where 5,025 were successfully mapped across all 12 chromosomes. We then filtered the markers to eliminate those with a minor allele frequency (MAF) below 0.01 or exceeding a 20% genotype missing rate. This resulted in a set of 4,706 high-quality SNP markers that were used for subsequent population structure and GWAS analyses.

The SNPs were uniformly distributed throughout the genome, with an average of 392 SNPs, ranging from 252 to 598, per chromosome. The nonsynonymous/synonymous (*Ka/Ks*) ratio of 0.71 indicated potential purifying or positive selection, while the transition-to-transversion (*Ts/Tv*) ratio of 3.94 (A/C: 476, A/G: 1871, C/T: 1882, G/T: 477) suggested a higher prevalence of transitions than transversions. The elevated *Ts/Tv* ratio was possibly influenced by DNA repair mechanisms or evolutionary constraints (Strandberg and Salter, 2004). The overall genotypic heterozygosity among the 110 accessions was 27.4%, which is consistent with previous reports (Berdugo-Cely et al., 2023).

**Figure 3:**
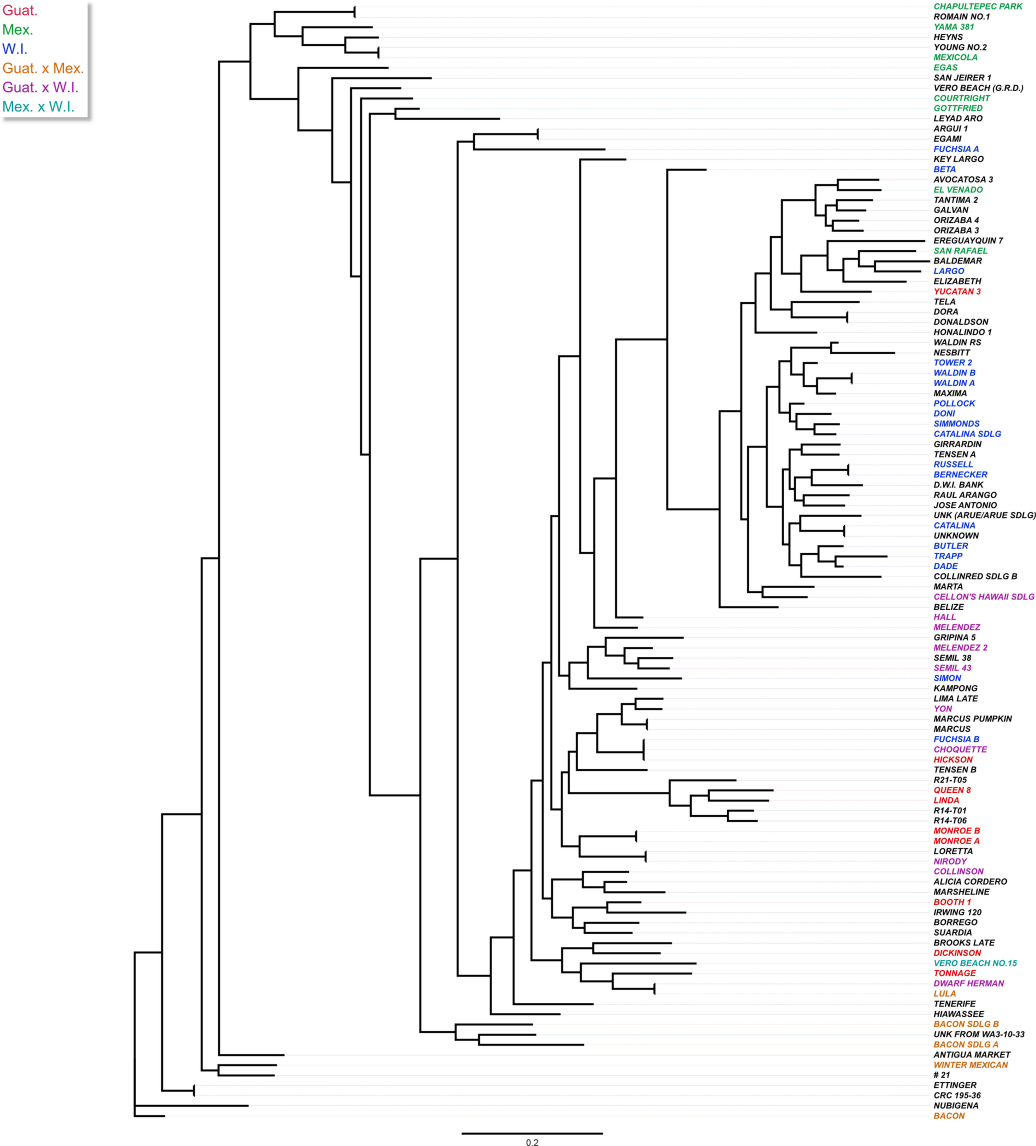
Maximum-likelihood (ML) tree showing the phylogenetic relationship among 110 avocado accessions based on 4,706 SNP markers. The tree was built by RAxML using the GTRGAMMA model and supported by 100 bootstrap replicates after MAFFT alignment. The colors show the different avocado races based on the UC Riverside Avocado Variety Database (https://avocado.ucr.edu/avocado-variety-database, accessed on 24 October 2023) and the Variety Database (http://www.avocadosource.com/AvocadoVarieties/QueryDB.asp , accessed on 24 October 2023), with Guatemalan in red, Mexican in green, and West Indian in blue. Hybrids are depicted with Guat. x Mex. in orange, Guat. x W.I. in purple, and Mex. x W.I. in cyan. Accessions with no race reported are left uncolored.

A maximum-likelihood phylogenetic tree was constructed based on the 4,706 SNP markers to illustrate the evolutionary relationships among the 110 accessions (**Figure 3**). We found that avocado genotypes from the same race or hybrids with similar genetic compositions were closely related, and tended to cluster together in the tree, with only a few outliers, although each race contained many subclades. The Guatemalan and West Indian races were overall more closely related than the Mexican race among the selected avocado accessions. This finding was further supported by the pairwise *F*_ST_ analysis conducted among the three populations (**Table 1**). The Mexican and West Indian races were most distantly related in the phylogenetic tree. Hybrid varieties appeared to occupy an intermediate position in the evolutionary relationships. Additionally, the analysis suggested that hybrid varieties between the Mexican and West Indian races were relatively rare in our collection.

**Table 1:** Analysis of molecular variance (AMOVA) for 110 avocado accessions grouped into three clusters.

Source	df	SS	MS	Est. Var.	%
Among Pops	2	30394.986	15197.493	280.679	24%
Within Pops	217	189369.469	872.670	872.670	76%
Total	219	219764.455		1153.349	100%

df, degree of freedom; SS, sum of squares; MS, mean squares; Est. Var., estimated variance component; %, percentage of genetic variation. Fixation indices and p-values: F_ST_: 0.243, P < 0.001.

**Figure 4:**
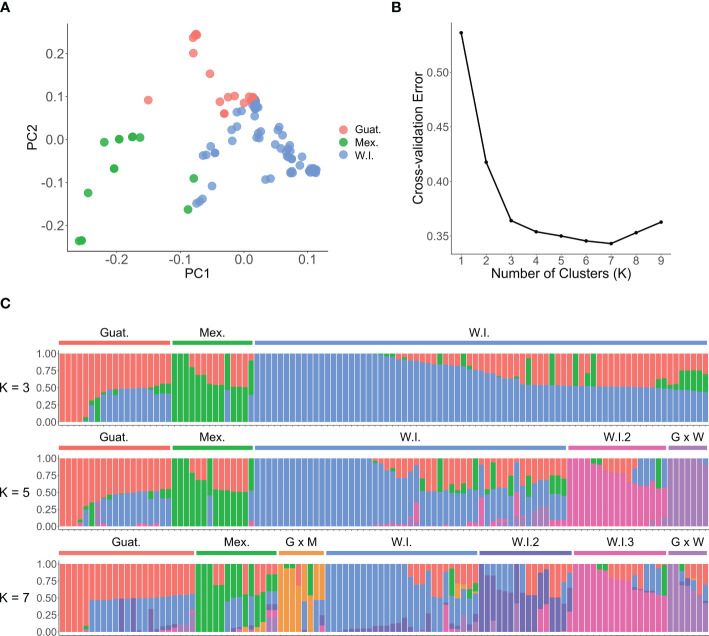
Population structure and principal component analysis (PCA) of 110 avocado accessions. **(A)** PCA scatter plot based on the first two components shows three groups, consistent with the three avocado races: Guatemalan (red), Mexican (green), and West Indian (blue). **(B)** The optimal number of genetic clusters K inferred from the ADMIXTURE cross-validation error. **(C)** The population structure plot from the ADMIXTURE analyses with K = 3, K = 5 and K = 7. The plot abbreviates Mexican as Mex. or M., West Indian as W.I. or W., Guatemalan as Guat. or G.

Based on the first two components, the PCA plot (**Figure 4A**) grouped avocado genotypes into three clusters. These clusters corresponded to the three ecotypes of their origins: Guatemalan (Guat.), Mexican (Mex.), and West Indian (W.I.). There was considerable overlap between the clusters, indicating the hybrid nature of many cultivars. An ADMIXTURE analysis of the 110 accessions revealed an optimal population number of K = 7 (**Figure 4B**). This resulted in three subclusters within the West Indian groups, and hybrids between different races were also regarded as new groups. However, there was no significant difference in cross-validation error rates between K = 3 and K = 7, pointing to the complex hybrid nature of many avocado cultivars. Dividing the 110 accessions into three populations generally aligned with the three avocado races, with only a few exceptions (**Figure 4C**; **Supplementary Table S1**). For example, ‘FUCHSIA B’, designated as a W.I. ecotype, grouped with other Guat. varieties with predicted probabilities (Guat. 51.2% and W.I. 48.8%). Also, these analyses revealed that FUCHSIA B is genetically identical to Choquette, alluding to possible mislabeling. ‘GOTTFRIED’ (Mex. 47.4% and W.I. 52.6%) and ‘COURTRIGHT’ (Mex. 46.2% and W.I. 53.8%), classified as Mex. ecotypes, and ‘YUCATAN 3’ (Guat. 23.9% and W.I. 76.1%) and ‘KEY LARGO’ (Guat. 48.4% and W.I. 51.6%), designated as Guat. ecotypes, were all erroneously classified into the W.I. group. This misclassification could be attributed to various factors, such as labelling, sampling or genotyping errors, historically incorrect race classification, or a limited number of SNPs considered in the structural analyses.

### Analysis of molecular variance and genetic diversity

The analysis of molecular variance (AMOVA) quantified the genetic differentiation between and within the three defined groups (**Table 2**). The study revealed that most (76%) of the observed genetic variation stemmed from differentiation between individuals within the populations. Furthermore, 24% of the molecular variation was observed between the three groups, indicating significant differentiation, and supporting the population structure results presented in **Figure 4C**.

**Table 2:** Population genetics parameters among three avocado ecotypes based on 4706 SNP genotypes.

Population 1	Population 2	*F*_ST_	*Nm*	Nei’s unbiased genetic identity
Guatemalan	Mexican	0.212	0.929	0.805
Guatemalan	West Indian	0.140	1.536	0.863
Mexican	West Indian	0.302	0.578	0.777

F_ST_, Fixation Index; Nm, Effective Migration Rate.

The expected heterozygosity (*H_E_*) values were calculated for the three avocado populations, providing insights into their genetic diversity. Among these populations, Guat. exhibited the highest expected heterozygosity (*H_E_* = 0.127), indicating relatively greater genetic diversity within this population. In comparison, Mex. showed the lowest expected heterozygosity (*H_E_* = 0.104) and W.I., despite its large population size, demonstrated an intermediate expected heterozygosity (*H_E_* = 0.118).

Population genetic differentiation was evaluated using pairwise fixation index (*F*_ST_) values, which range from 0 (no differentiation) to 1 (complete differentiation) (**Table 1**). Varying degrees of genetic differentiation were observed with the weighted Weir and Cockerham *F*_ST_ values accounting for differences in sample size. A moderate level of genetic differentiation was observed between the Guatemalan and Mexican populations (*F*_ST_ = 0.212), indicating significant genetic differences between the two groups. Similarly, the Guatemalan and West Indian populations showed lower but still significant levels of differentiation (*F*_ST_ = 0.140). The highest level of differentiation was observed between the Mexican and West Indian populations (*F*_ST_ = 0.302), indicating substantial genetic divergence between these two groups. These results are generally consistent with previous reports of strong population structure in avocado (Berdugo-Cely et al, 2023), except for the much lower pairwise *F*_ST_ between the Guatemalan and West Indian populations in our study. The measure of gene flow, *Nm*, between the Guatemalan and West Indian populations was 1.536, the highest among the three pairs, indicating relatively higher gene flow between these populations than others. Additionally, the highest Nei’s unbiased genetic identity value of 0.863 was overserved between the Guatemalan and West Indian populations, indicating a greater genetic similarity between these two groups. In contrast, the lowest genetic identity of 0.777 was found between the Mexican and West Indian populations, suggesting a relatively higher divergence in between these two groups.

The linkage disequilibrium (LD) among the 4,706 SNPs was estimated for three populations as illustrated in **Supplementary Figure S1**. The correlation coefficients (*r* ^2^) for all three populations within a 5 Mb distance ranged between 0.1 and 0.2, indicating a generally low correlation between these SNP markers and a high genetic diversity within the studied populations. Among the three populations, the Mexican race showed the highest LD coefficient value, with a mean of 0.195 at 1.91 Mb. Conversely, the West Indian race displayed the lowest LD coefficient, with a mean of 0.099 at 1.50 Mb, suggesting a higher level of recombination contributing to the genetic diversity. The Guatemalan race fell between these two, with an intermediate LD coefficient of 0.162 at 2.39 Mb. Overall, these results indicate that the SNP data is suitable for GWAS analysis.

### Identification of markers for fruit quality traits

**Figure 5:**
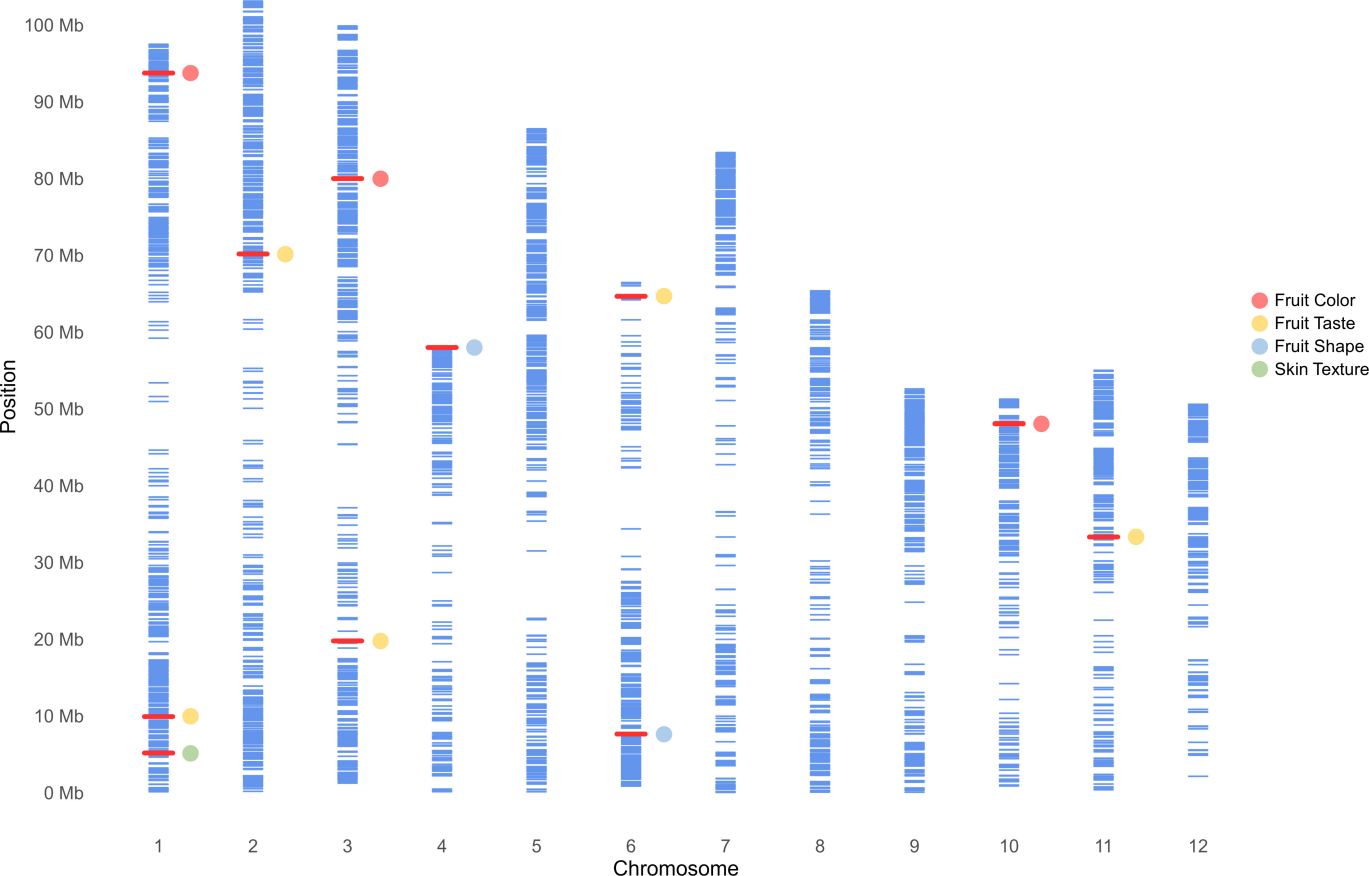
Physical map showing the distribution of 4,706 SNPs across the genome of 110 avocado accessions, generated using a 6k SNP array. Physical locations are in millions of base pairs (Mb) based on the 12 chromosomes of the ‘Hass’ reference genome (GCA_029852735.1; Nath et al., 2022). Each blue bar represents a SNP, while red bars highlight markers associated with fruit quality traits identified in this study.

GWAS was carried out using 4,706 SNP markers, which were derived from transcriptomic data and were distributed throughout the avocado genome. Two models, GLM (PCA) and MLM (PCA + K), were used to explore their associations with fruit quality traits in 110 avocado accessions (**Supplementary Tables S4**, **S5**). Q-Q plots to evaluate the distribution of *p*-values of GWAS results and to assess model performance and statistical validity are shown in **Supplementary Figures S2**–**S4**. The MLM models generally exhibited better *p*-value distribution than the GLM models, particularly in the GWAS for red skin color and clavate fruit shape. The MLM model demonstrated a good *p*-value distribution with minimal inflation, and the markers associated with the phenotype showed significantly higher than the expected -log_10_(*p*) values. Only 6 markers associated with fruit color and shape were identified using the MLM model using a strict Bonferroni threshold. Therefore, to avoid neglecting important candidate regions, weaker thresholds of *p* = 0.0001 and 0.0005 were considered for fruit taste and skin texture related traits. As a result, a total of 12 SNPs from eleven genomic regions were eventually identified and supported by both models (**Figure 5**, **Table 3**). All markers identified by the MLM (PCA + K) model were also identified by the GLM model, but not vice versa. These markers were located on seven chromosomes, specifically chr.1, 2, 3, 4, 6, 10, and 11. The *p*-values according to the MLM model ranged from 6.93E-09 to 5.45E-04 for markers SHRSPaS005193 and SHRSPaS006369, respectively. The phenotypic variation *R* ^2^ varied from 14.84% to 43.96%. Further, the allele effects ranged from -1.93 (I) to 2.04 (G) for markers SHRSPaS005193 and SHRSPaS002116, respectively (**Supplementary Table S6**).

**Table 3:** List of markers associated with avocado fruit quality traits using MLM (PCA + K) model.

Trait	SNP	Chr	Position	*P*-value (MLM)	*R^2^*	Gene Name	Product
Green skin color	SHRSPaS002116	1	93,731,507	5.94E-06	0.22	*MRB53_003934*	Pheophytinase
Red skin color	SHRSPaS005193	10	48,081,345	6.93E-09	0.44	*MRB53_031935*	Regulatory photoreceptor
Red skin color	SHRSPaS005886	10	48,041,147	6.93E-09	0.44	*MRB53_031935*	Regulatory photoreceptor
Red skin color	SHRSPaS006476	3	79,987,394	5.65E-06	0.26	*MRB53_011779*	Geranylgeranyl transferase
Skin texture	SHRSPaS005282	1	5,169,081	3.26E-04	0.16	*MRB53_000404*	Auxin response factors (ARFs)
Pyriform shape	SHRSPaS006814	4	57,984,086	6.55E-06	0.26	*MRB53_015427*	Developmentally regulated
Clavate shape	SHRSPaS006438	6	7,636,668	4.36E-06	0.26	*MRB53_019708*	Calcium-binding protein
Nutty taste	SHRSPaS005469	3	19,765,354	4.61E-04	0.15	*MRB53_009721*	TRANSPARENT TESTA
Nutty taste	SHRSPaS006369	1	9,907,663	5.45E-04	0.15	*MRB53_000723*	Regulation of actin nucleation
Creamy taste	SHRSPaS006954	11	33,301,647	1.70E-05	0.23	*MRB53_033129*	E3 ubiquitin protein ligase
Watery taste	SHRSPaS005589	6	64,653,962	1.53E-04	0.18	*MRB53_021754*	Inositol monophosphatase family
Bitter taste	SHRSPaS005603	2	70,167,527	5.51E-05	0.20	*MRB53_006642*	pectinesterase

Candidate genes were determined based on functional relevance within 100 kb windows centered on each marker. R^2^ = coefficient of determination, a statistical measure, quantifies the proportion of variation in phenotype that can be explained by the genetic marker. This calculation was performed using TASSEL 5.0.

**Figure 6:**
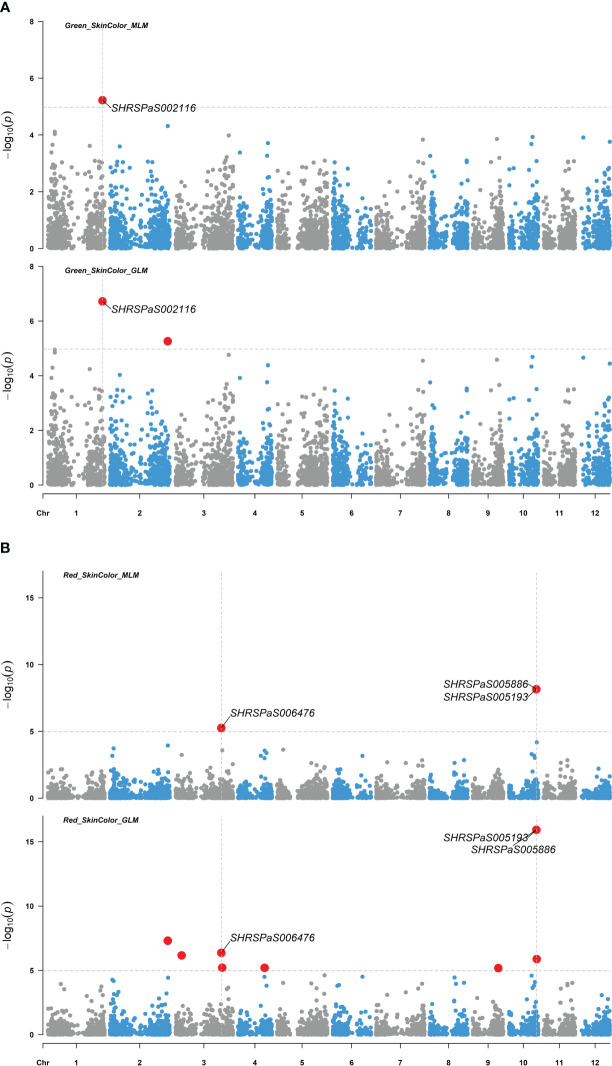
Genome-wide association studies of avocado fruit skin color. Manhattan plots depicting the association between SNP markers across the 12 chromosomes of the ‘Hass’ reference genome (GCA_029852735.1) and fruit skin color intensity using 110 avocado (*Persea americana*) accessions. Results from both the MLM (PCA + K) and GLM (PCA) models are compared, with significant SNPs highlighted by enlarged red dots. The significance threshold after Bonferroni-correction is indicated by gray horizontal dashed lines at -log_10_(*p*) = 4.97. **(A)** SNP distribution based on association with green fruit color intensity. **(B)** SNP distribution based on association with red fruit color intensity.

**Figure 7:**
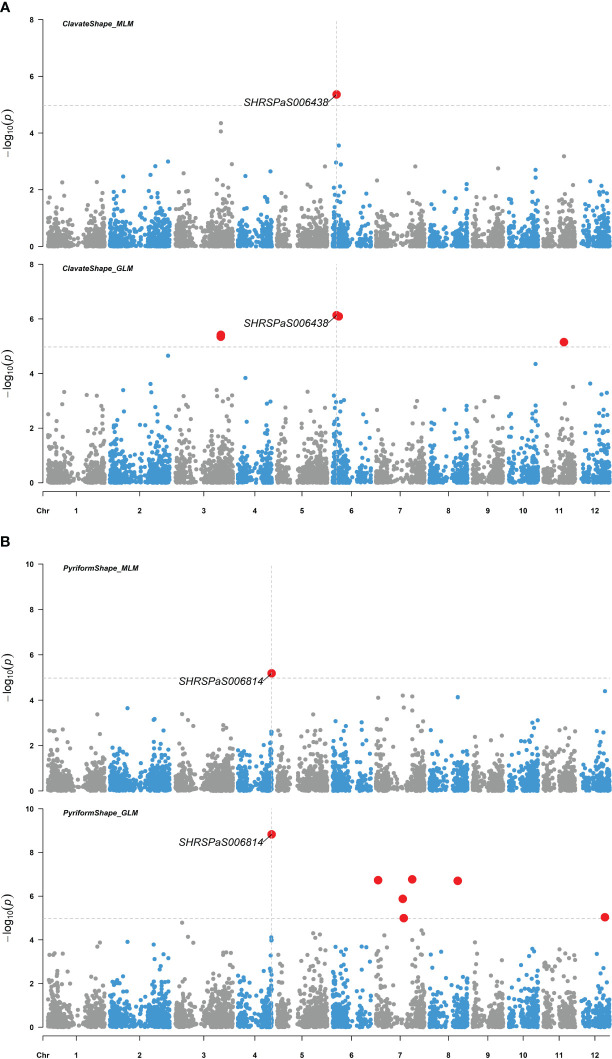
Genome-wide association studies of avocado fruit shape. Manhattan plots showing the association between SNP markers and fruit shapes of 110 avocado (*Persea americana*) collections, utilizing both the MLM (PCA + K) and the GLM (PCA) models. Significant SNPs are highlighted by enlarged red dots, with the significance threshold after Bonferroni-correction indicated by gray horizontal dashed lines at -log_10_(*p*) = 4.97. **(A)** The association with pear-like pyriform shape compared to the other shapes. **(B)** The association with elongated clavate shape compared to the other shapes.

Four markers showed significant association with fruit skin colors, with -log_10_(*p*) values greater than or equal to 4.97. One marker on chromosome 1 was found to be associated with green skin color, which explained 21.6% of the variation. Three markers were related to red skin color: one on chromosome 3 and two adjacent on chromosome 10 with 40,198 bp between them (**Figure 6**). Both markers on chromosome 10 were significantly associated with red skin color under the MLM model (-log_10_(*p*) = 8.16) and GLM (-log_10_(*p*) = 15.92), explaining 44.0% and 48.6% of the variation, respectively. Two markers on chromosomes 4 and 6 were significantly linked to pyriform and clavate fruit shapes, supported by high -log_10_(*p*) values (**Figure 7**).

**Figure 8:**
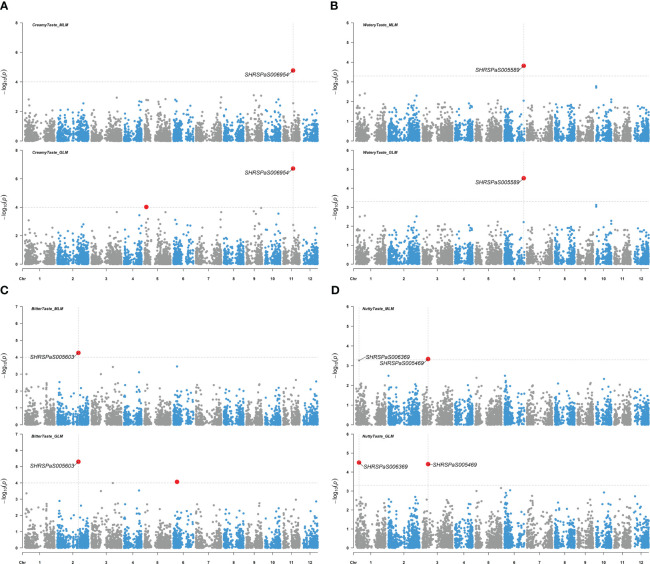
Genome-wide association studies for avocado fruit taste traits. Manhattan plots showing the association between SNP markers and fruit taste data in avocados, utilizing results from both the MLM (PCA + K) and the GLM (PCA) models. Gray horizontal dashed lines represent soft significance thresholds of -log_10_(*p*) with *p* = 0.0001 or *p* = 0.0005. Significant SNPs are denoted by enlarged red dots. **(A)** The association with creamy fruit taste compared to the other tastes. **(B)** The association with watery fruit taste compared to the other tastes. **(C)** The association with bitter fruit taste compared to the other tastes. **(D)** The association with nutty fruit taste compared to the other tastes.

**Figure 9:**
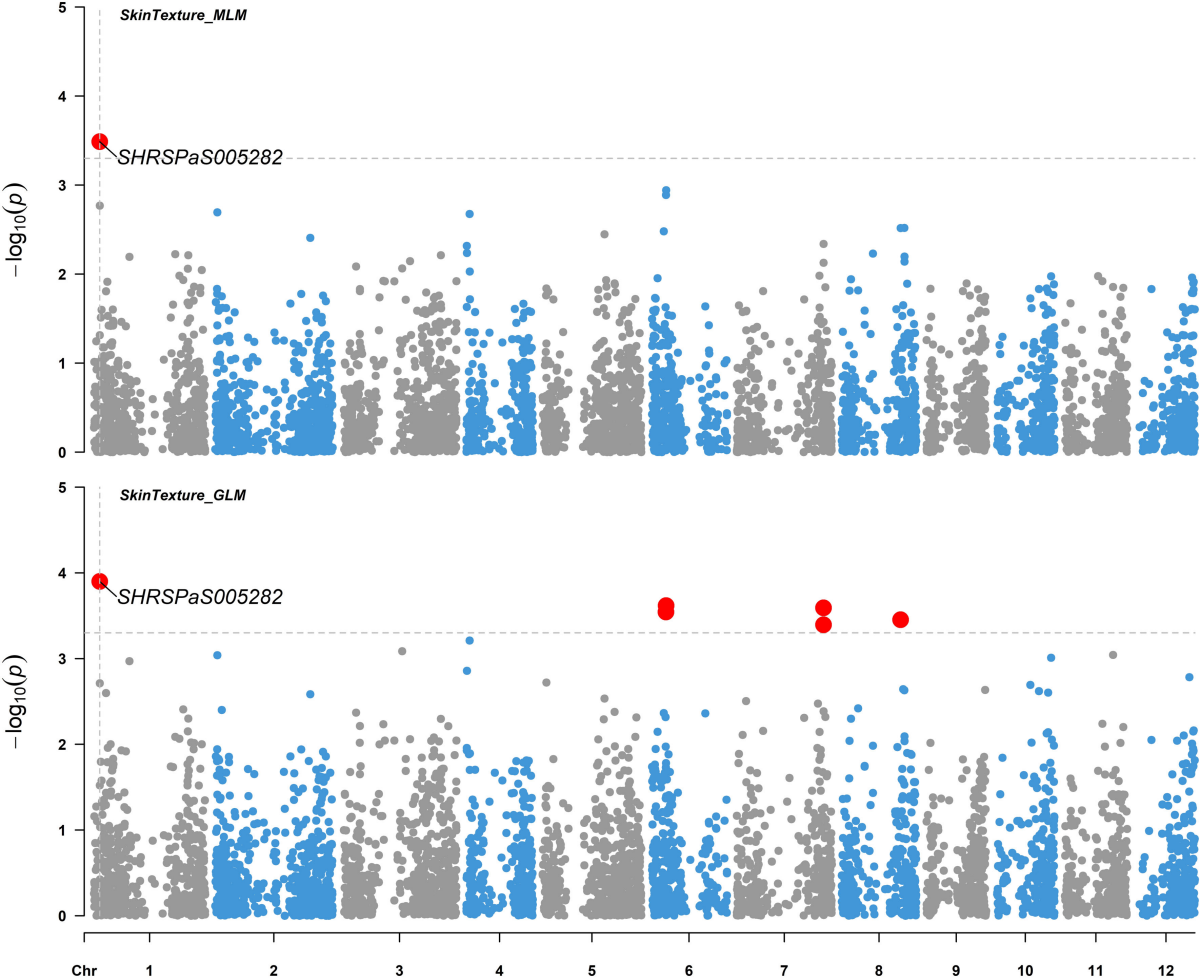
Genome-wide association studies for avocado fruit skin texture. Manhattan plots illustrating the association between SNP markers and fruit skin texture data from 110 avocado (*Persea americana*) collections. Results from both the MLM (PCA + K) model (upper panel) and GLM (PCA) model (lower panel) using 4706 SNP markers across the 12 chromosomes of the ‘Hass’ reference genome (GCA_029852735.1) are aligned for comparison. The gray horizontal dashed lines indicate a soft significance threshold of -log_10_(*p*) when *p* = 0.0005. Significant SNPs are represented by enlarged red dots. The fruit skin texture is divided into three categories: smooth, rough, and very rough.

Five markers on chromosomes 1, 2, 3, 6, and 11, which displayed higher -log_10_(*p*) values than the rest of SNPs but were below the -log_10_(*p*) = 4.97 threshold, showed moderate associations with various fruit tastes (**Figure 8**), explaining 14.8 - 25.3% of the variation. Additionally, one maker was found to be associated with skin texture with *p* value < 0.0005 (**Figure 9**) and explained 16.4% of the variation in skin texture by MLM model.

It is important to note that in addition to the MLM model, the GLM model identified additional markers, which, due to the quantitative nature of the analyzed traits, might be important. The GLM model identified 26 significant markers associated with fruit quality traits, with a *p*-value lower than 1.06E-05 (0.05/4,706) (**Supplementary Table S5**). These markers were distributed across all avocado chromosomes except chromosome 5, with the highest number (5) located on chromosome 3 and the lowest number (1) on chromosomes 1, 9, and 12. The *p*-value ranged from 1.19E-16 to 1.02E-05 for markers SHRSPaS005886 and SHRSPaS002716, respectively. The phenotypic variation *R* ^2^ ranged from 17.50% to 48.58%.

### Gene enrichment analyses in the vicinity of significant markers

Annotations of genes within a 200 kb window, with an average LD (*r* ^2^) of approximately 0.2, centered on each of the 12 markers, identified a total of 136 genes. Candidate genes for fruit quality traits were inferred based on the functions of these 136 genes (**Table 3**; **Supplementary Table S6**). Genes encoding pheophytinase (*MRB53_003934*) and regulatory photoreceptors (*MRB53_031935* and *MRB53_011779*) were implicated in controlling fruit color. The developmentally regulated gene (*MRB53_015427*) and calcium-binding protein encoding gene (*MRB53_019708*) were associated with fruit pyriform and clavate shapes. Five genes were linked to fruit tastes, including the protein TRANSPARENT TESTA gene (*MRB53_009721*) and the regulation of actin nucleation gene (*MRB53_000723*) for nutty taste, the E3 ubiquitin protein ligase gene (*MRB53_033129*) for creamy taste, the Inositol monophosphatase family gene (*MRB53_021754*) for watery taste, and the pectinesterase gene (*MRB53_006642*) for bitter taste. The auxin response factors (ARFs) gene (*MRB53_000404*) was associated with fruit skin texture.

**Figure 10:**
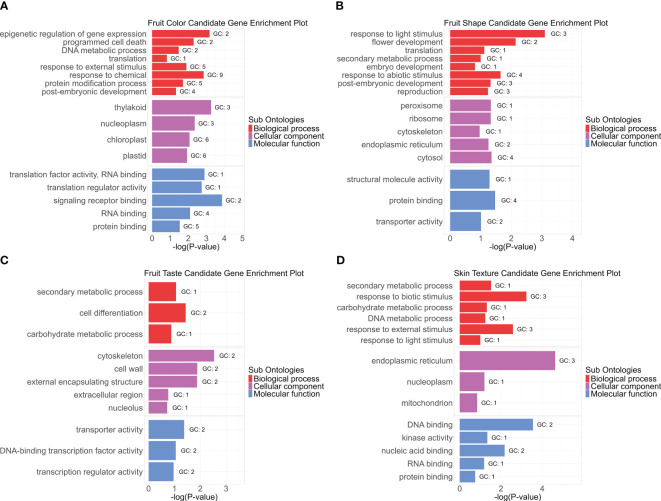
Gene Ontology (GO) enrichment analysis for avocado fruit quality traits. The enrichment includes three sub-ontologies: biological process (BP), cellular component (CC), and molecular function (MF). Each panel **(A-D)** represents one category of fruit quality trait: **(A)** fruit color, **(B)** fruit shape, **(C)** fruit taste, and **(D)** fruit skin texture. The GO terms are plotted against significance -log(*p*-value), sorted within each subontology group by enrichment score from high at the top to low at the bottom. Only enrichment scores greater than 1.5 are displayed. “GC” represents the hit gene counts.

Gene Ontology (GO) enrichment analysis of 136 candidate genes associated with fruit color, shape, taste, and skin texture revealed significantly represented pathways (**Figure 10**; **Supplementary Table S7**). Notably, the thylakoid as well as plastids and chloroplasts emerged as the most enriched cellular component term for fruit color. Thylakoids are membrane-bound compartments within chloroplasts, which house chlorophyll and other pigments critical for photosynthesis (Juhler et al., 1993; Allen, 2004; Collins et al., 2012). Epigenetic regulation of gene expression and response to chemicals stood out as highly enriched biological processes, while translation factor activity, RNA binding, and translation regulator activity were prominent molecular functions. Regarding fruit shape, biological processes related to light stimulus response and flower development were enriched. Cellular components such as peroxisomes and ribosomes played key roles, with structural molecule activity as the primary molecular function. For taste-related aspects, the cytoskeleton and cell wall were the most enriched terms in cellular components. Secondary metabolic processes dominated the biological functions, while transporter activity and DNA-binding transcription factor activity were notable molecular functions.

**Figure 11:**
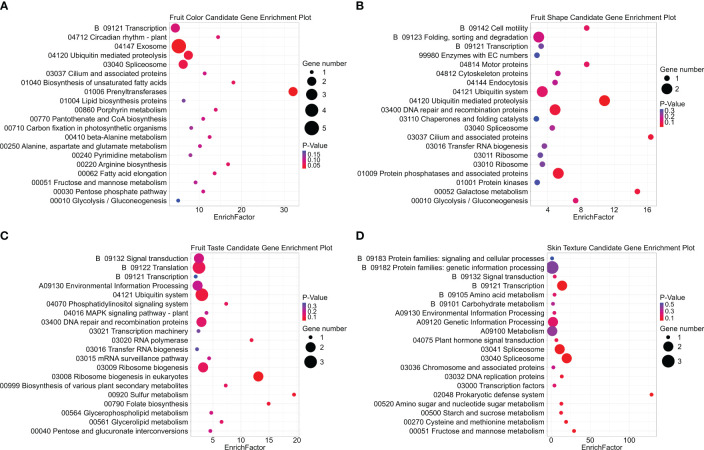
KEGG pathway enrichment analysis displaying the top 20 pathways for each fruit trait candidate gene group. Dot size represents the count of gene hits, while dot color indicates the significance of enrichment, with red denoting significant enrichment and purple indicating weaker significance. Subplots include **(A)** fruit color, **(B)** fruit shape, **(C)** fruit taste, and **(D)** fruit skin texture candidate gene groups.

Kyoto Encyclopedia of Genes and Genomes (KEGG) pathway enrichment analysis was conducted on the same gene set for fruit color, shape, taste, and skin texture (**Figure 11**; **Supplementary Table S8**). The prenyltransferases pathway exhibited the highest enrichment factor for fruit color. Although prenyltransferases may not directly influence fruit color, they are involved in the early steps of carotenoid biosynthesis, by catalyzing the transfer of prenyl groups to the precursor molecules (Liu et al., 2013; Winkelblech et al., 2015). Carotenoids are the primary pigments responsible for yellow, orange, and red fruit colors (Rodríguez-Concepción et al., 2018; Barja & Rodríguez-Concepción, 2021). The cilium and associated protein pathway showed significant enrichment for fruit shape. Cilia are microtubule-based organelles found on the surface of many cell types, including those involved in plant development (Senatore et al., 2022). Previous studies linked the overexpression of Microtubule-Associated Proteins SlMAP70 or SlIQD21a to elongated fruit shapes in tomatoes(*Solanum lycopersicum* L.) (Han et al., 2008; Bao et al., 2023). Additionally, sulfur metabolism emerged as the most enriched pathway for fruit taste. This pathway is related to methionine synthesis, which serves as the precursor for ethylene, a vital plant hormone regulating fruit ripening and flavor development (Baur and Yang, 1972; Wawrzyńska et al., 2015).

## Discussion

In this study, a genome-wide association study was conducted to uncover the genetic basis of fruit quality traits in 110 avocado accessions, leveraging high-quality SNP markers and phenotypic data. Prior to GWAS analyses, the population structure of these trees was analyzed systematically. Both ADMIXTURE-based population structure analysis and principal component analysis (PCA) consistently classified the 110 avocado accessions into three distinct groups, aligned with the three recognized races of avocado: Mexican, Guatemalan and West Indian (Bergh and Ellstrand, 1986; Chen et al., 2008). The classification system was used to determine the genetic makeup of each accession, especially for cultivars with unknown origins or unclear pedigrees. Phylogenetic analysis also exhibited three primary clusters that corresponded to the three ecotypes of avocados. Consistent with previous studies, most mixed-race hybrids situated between the three primary races (Schnell et al., 2003; Gross-German and Viruel, 2013; Ge et al., 2019; Rendon-Anaya et al., 2019; Rubinstein et al., 2019; Talavera et al., 2019; Wienk et al., 2022; Berdugo-Cely et al., 2023). However, contrary to several earlier studies that reported a closer relationship between Guatemalan (Guat.) and Mexican (Mex.) races than West Indian (W.I.) races (Gross-German and Viruel, 2013; Ge et al., 2019; Wienk et al., 2022; Berdugo-Cely et al., 2023), our observations indicate a closer relationship between Guat. and W.I. than Mex. This may be related to the overrepresentation of Guatemalan x West Indian hybrids in our collection, leading to a high similarity between the two groups. These findings have implications for breeding programs and conservation efforts, ultimately contributing to the improved genetic integrity of avocado germplasm collections.

This finding aligns with studies based on plant morphological characteristics (Kopp, 1966), microsatellite markers (Ashworth and Clegg, 2003), and SNP analyses (Rubinstein et al., 2019). Inconsistencies among different studies indicate the complex genetics of avocado hybrids. This discrepancy may be caused by differences and/or misidentification of cultivars or limited sample sizes in some studies. The West Indian and Mexican races exhibited the greatest divergence, with the Guatemalan race positioned between them in the evolutionary relationship. This finding was further supported by pairwise fixation index (*F*_ST_) analysis, which demonstrated that Guatemalan and West Indian races had the lowest *F*_ST_ of 0.140, while West Indian and Mexican races had the highest *F*_ST_ of 0.302. Our study reported a lower pairwise *F*_ST_ value between Guatemalan and West Indian races compared to the findings of Berdugo-Cely et al. (2023). The intriguing difference can be attributed to the substantial representation of the West Indian population in our study, encompassing numerous hybrids with intricate genetic compositions. Additionally, our analysis accounted for sample size, a factor that could contribute to the observed reduction in *F*_ST_ values. Lower levels of LD (*r*2) were observed in the West Indian group, suggesting higher differentiation and diversity within this group compared to the other two groups. However, this may also be influenced by the larger sample size of the West Indian accessions used in this study.

In this study, twelve markers in eleven regions significantly associated with different fruit quality traits of avocados were identified. Candidate genes were found based on their functional relevance, although further functional validation is needed. Among these genes, *MRB53_003934* was found to be related to the green skin color of avocados. The gene encodes pheophytinase (PPH), a phytol hydrolase that plays an essential role in chlorophyll degradation. As a photosynthetic pigment, chlorophyll is widely distributed in leaves and fruits, especially in early-stage fruits, being responsible for their greenish color. As fruits ripen, the degradation of chlorophyll leads to the de-greening of the fruit color, commonly seen in apples, citruses, and mangoes (Yin et al., 2016; Chen et al., 2022; Gorfer et al., 2022). In cultivars with green retention fruits, they exhibited a reduced degradation of chlorophyll pigments (Hornero-Méndez and Mínguez-Mosquera, 2002). *MRB53_031935* and *MRB53_011779* were identified as the genes controlling the red fruit color in avocados. *MRB53_031935* encodes phytochrome B (PHYB), the primary red-light photoreceptor in plants. Its forms reversibly interconvert by light: the Pr form absorbs maximally in 660 nm (red light), while the Pfr form absorbs maximally at the 730 nm wavelengths (far-red light) (Grimm et al., 1993). One of the main contributors to red fruit skin color is the accumulation of anthocyanins (Denoyés-Rothan et al., 2023). Phytochrome was found to mediate the synthesis of anthocyanin and other pigments in plants (Lange et al., 1971; Huub et al., 1997). Overexpression of *PHYB2* results in the fruit color change at the breaker stage of tomato (Alves et al., 2020). *MRB53_011779* encodes geranylgeranyl transferase (GGTase). Geranylgeranyl diphosphate (GGPP) serves as the precursor for carotenoid biosynthesis, and the latter is a lipophilic natural pigment giving plant tissues a red, yellow, or orange color (Rodríguez-Concepción et al., 2018; Barja & Rodríguez-Concepción, 2021). The accumulation of *β*-carotene explains the yellowish color of Golden Rice (*Oryza sativa*) (Schaub et al., 2005). GGTase is an enzyme involved in the post-translational modification of proteins by attaching geranylgeranyl groups onto them. Although GGTase is not directly required in the synthesis of pigments (Rodríguez-Concepción and Gruissem, 1999), its activity can indirectly influence fruit color development by modulating cellular processes, signaling pathways, and gene expression networks associated with pigmentation during fruit development and ripening.

The developmentally regulated gene (*MRB53_015427*) and calcium-binding protein encoding gene (*MRB53_019708*) have been identified as candidate genes for determining the pyriform and clavate fruit shapes in avocados, respectively. In tomato, the pear shape is controlled by a single recessive locus called *ovate* (Wang et al., 2016). This gene causes a change in cell distribution in ovaries, resulting in an increase in cell number along the proximo-distal axis and a decrease in cell number along the mediolateral axis at the proximal end, ultimately forming a pear shape (Wu et al., 2018; Li et al., 2023). *MRB53_015427* encodes a developmentally regulated protein that plays a critical role in pattern formation and cell fate specification during the development of multicellular organisms (Dickmeis and Müller, 2005). Meanwhile, the gene *SUN* has been identified as the critical regulator of fruit elongation in tomatoes. *SUN* encodes a calmodulin-binding protein that facilitates the longitudinal division of fruit cells while concurrently inhibiting transverse division by modulating plant hormone abundance (Han et al., 2008; Wu et al., 2011). *MRB53_019708* encodes a calcium-binding protein, which also plays an important role in the calcium signaling pathway. However, further research is needed to determine whether these genes are involved in related pathways that determine fruit shape.

Five genes were identified as potentially associated with fruit flavors: *MRB53_009721* and *MRB53_000723* for nutty taste, *MRB53_033129* for creamy taste, *MRB53_021754* for watery taste, and *MRB53_006642* for bitter taste. The gene *MRB53_006642* encodes pectin esterase, an enzyme that modifies pectin, a complex polysaccharide found in plant cell walls. Unripe fruits usually contain higher levels of pectin, which breaks down during fruit ripening (Paniagua et al., 2014). The main contributor to fruit bitterness is limonin (Hasegawa et al., 1996), and pectin was found to increase the limonin solubility and cause bitterness in juice (Chandler, 1971). The gene *MRB53_033129* encodes an E3 ubiquitin-protein ligase, which plays a crucial role in protein ubiquitination, a key post-translational modification involved in protein degradation and regulation of cellular processes (Yang et al., 2021). E3 ubiquitin-protein ligase is important in regulating fruit ripening (Y. Wang et al., 2022), and may affect the taste of the fruit indirectly. The *MRB53_021754* gene encodes a protein belonging to the inositol monophosphatase family. In tomato, the myoinositol monophosphatase 3 gene, SlIMP3, is closely related to this family, which was found to be expressed at the highest expression level in fruit, controlling fruit development and quality. SIIMP3 acts as a bifunctional enzyme involved in the biosynthesis of ascorbic acid (AsA) and myoinositol. Zheng et al. (2022) demonstrated that overexpression of SlIMP3 in tomato could also increase cell wall thickness, improve fruit firmness, delay fruit softening, reduce water loss, and extend shelf life. Therefore, these may indirectly affect the moisture content and mouthfeel of the fruit. The gene auxin response factors (ARFs) (*MRB53_000404*) are significantly associated with the texture of fruit skin. These transcription factors regulate gene expression in response to the phytochrome auxin. While the impact of ARFs on fruit skin texture has been unknown, auxin signaling pathways have been linked with various aspects of fruit development and ripening. Auxin is known to regulate plant cell and cell wall expansion and to control cuticle formation (Masuda, 1990; Majda and Robert, 2018; Qiao et al., 2019).

This study has improved our understanding about the genetic basis of avocado’s fruit quality traits, including fruit color, shape, taste, and skin texture, by pinpointing associated genetic regions and candidate genes. Future research can enhance these findings by fine-mapping polymorphisms using high-resolution genome resequencing data and validating associations with these traits in segregating populations. Investigating gene expression levels in individuals with distinct characteristics or conducting gain- and loss-of-function experiments could shed light on the functional significance of these genes. The findings in this report have potential practical implications using marker-assisted selection and genomic selection in accelerating the breeding of fruit crops.

## Data Availability

The datasets presented in this study can be found in online repositories. The names of the repository/repositories and accession number(s) can be found in the article/**Supplementary Material**.
