# Supplementary material for: Comprehensive genetic diversity and genome-wide association studies revealed the genetic basis of avocado fruit quality traits
Source: Front Plant Sci. 2024 Aug 13;15:1433436. doi: 10.3389/fpls.2024.1433436 (PMC11347836; doi:10.3389/fpls.2024.1433436)
Supplement: Supplementary file 1 [file DataSheet1.pdf]

Supplementary Materials

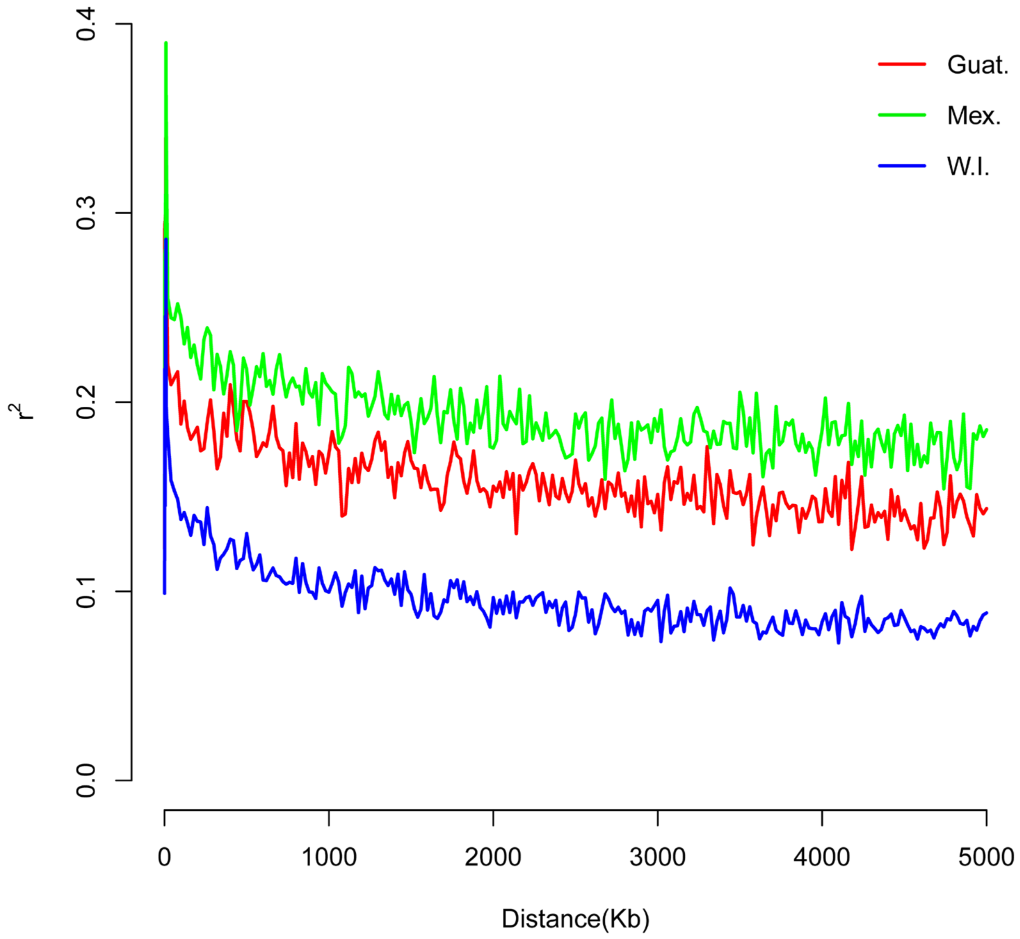

Figure S1. Genome-wide linkage disequilibrium (LD) decay plot drawn by PopLDdecay with squared correlation (r^2) plotted against genetic distance for SNP pairs. Bin1 and Bin2 window sizes were set to 1 kb and 20 kb, respectively, with a breakpoint at 10 kb.

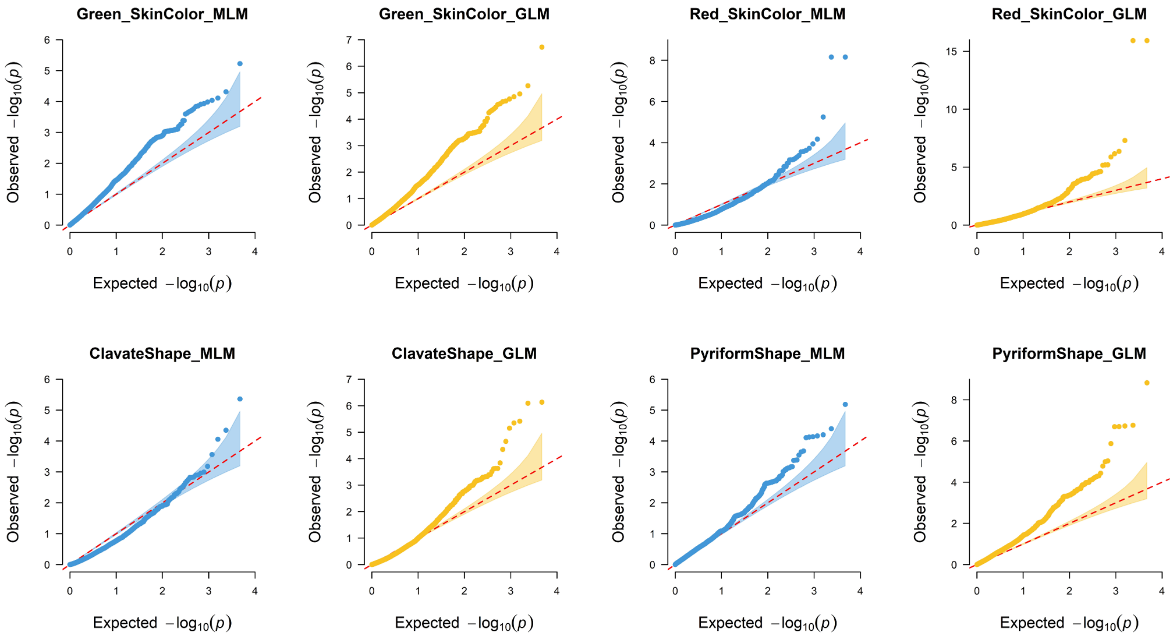

Figure S2. Quantile-Quantile (QQ) plots of the avocado fruit skin color and fruit shape genome-wide association studies using the General Linear Models (GLM) and Mixed Linear Models (MLM). Displayed are observed -log$_{10}$(*P*-value) versus expected -log$_{10}$(*P*-value). The shaded area represents the 95% confidence interval.

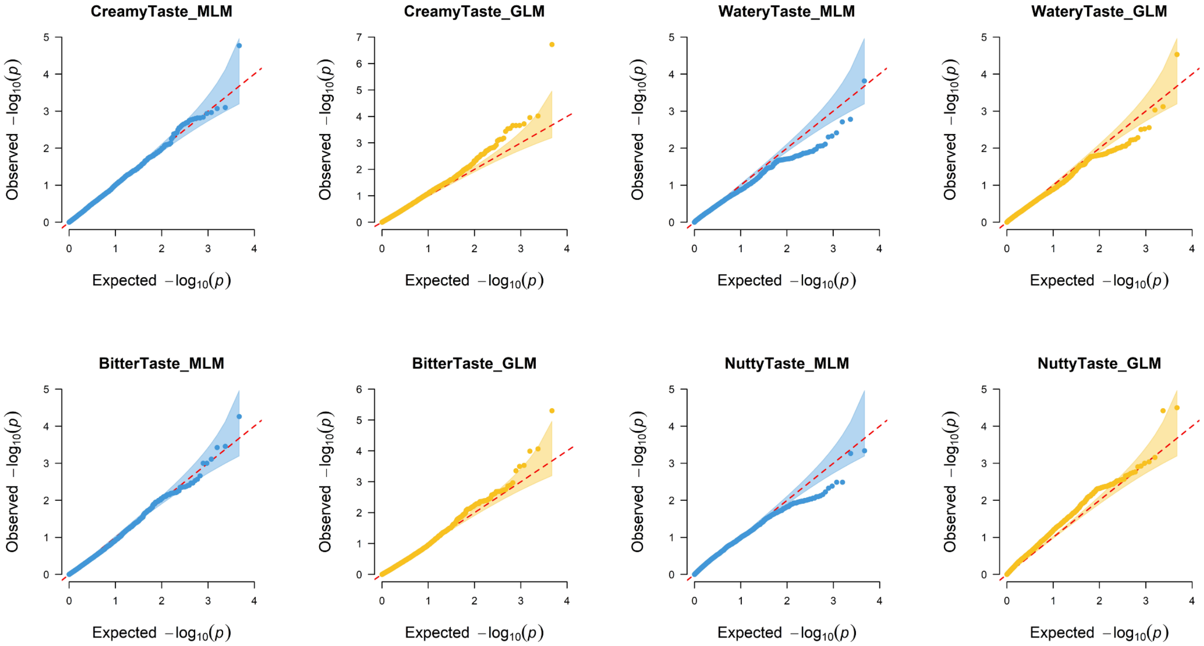

Figure S3. Quantile-Quantile (QQ) plots of the avocado fruit taste genome-wide association studies using the General Linear Models (GLM) and Mixed Linear Models (MLM). Displayed are observed -$\log_{10}$(P-value) versus expected -$\log_{10}$(P-value). The shaded area represents the 95% confidence interval.

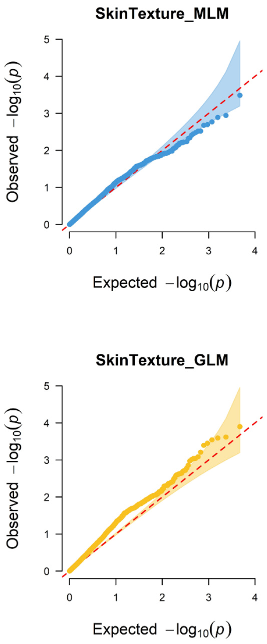

Figure S4. Quantile-Quantile (QQ) plots of the avocado skin texture association studies using the General Linear Models (GLM) and Mixed Linear Models (MLM). Displayed are observed -$\log_{10}$(P-value) versus expected -$\log_{10}$(P-value). The shaded area represents the 95% confidence interval.
